# Hematological, Micro-Rheological, and Metabolic Changes Modulated by Local Ischemic Pre- and Post-Conditioning in Rat Limb Ischemia-Reperfusion

**DOI:** 10.3390/metabo11110776

**Published:** 2021-11-13

**Authors:** Csaba Korei, Balazs Szabo, Adam Varga, Barbara Barath, Adam Deak, Erzsebet Vanyolos, Zoltan Hargitai, Ilona Kovacs, Norbert Nemeth, Katalin Peto

**Affiliations:** 1 Department of Traumatology and Hand Surgery, Faculty of Medicine, University of Debrecen, Bartok Bela ut 2-26, H-4031 Debrecen, Hungary; korei.csaba@med.unideb.hu; 2 Department of Operative Techniques and Surgical Research, Faculty of Medicine, University of Debrecen, Moricz Zsigmond u. 22, H-4002 Debrecen, Hungary; balazsszabo929@gmail.com (B.S.); varga.adam@med.unideb.hu (A.V.); barath.barbara@med.unideb.hu (B.B.); deak.adam@med.unideb.hu (A.D.); vanyolos@med.unideb.hu (E.V.); kpeto@med.unideb.hu (K.P.); 3 Doctoral School of Clinical Medicine, University of Debrecen, Nagyerdei krt. 98, H-4032 Debrecen, Hungary; 4 Clinical Center, Pathology Unit, Kenezy Campus, University of Debrecen, Bartok Bela ut 2-26, H-4031 Debrecen, Hungary; z.hargy@gmail.hu (Z.H.); dr.kovacs.ilona@kenezy.unideb.hu (I.K.)

**Keywords:** limb ischemia-reperfusion, hemorheology, metabolites, ischemic pre-conditioning, ischemic post-conditioning

## Abstract

In trauma and orthopedic surgery, limb ischemia-reperfusion (I/R) remains a great challenge. The effect of preventive protocols, including surgical conditioning approaches, is still controversial. We aimed to examine the effects of local ischemic pre-conditioning (PreC) and post-conditioning (PostC) on limb I/R. Anesthetized rats were randomized into sham-operated (control), I/R (120-min limb ischemia with tourniquet), PreC, or PostC groups (3 × 10-min tourniquet ischemia, 10-min reperfusion intervals). Blood samples were taken before and just after the ischemia, and on the first postoperative week for testing hematological, micro-rheological (erythrocyte deformability and aggregation), and metabolic parameters. Histological samples were also taken. Erythrocyte count, hemoglobin, and hematocrit values decreased, while after a temporary decrease, platelet count increased in I/R groups. Erythrocyte deformability impairment and aggregation enhancement were seen after ischemia, more obviously in the PreC group, and less in PostC. Blood pH decreased in all I/R groups. The elevation of creatinine and lactate concentration was the largest in PostC group. Histology did not reveal important differences. In conclusion, limb I/R caused micro-rheological impairment with hematological and metabolic changes. Ischemic pre- and post-conditioning had additive changes in various manners. Post-conditioning showed better micro-rheological effects. However, by these parameters it cannot be decided which protocol is better.

## 1. Introduction

Acute limb ischemia still remains a great challenge in clinical practice. In connection with traumatic injuries, vascular and orthopedic surgery the blood supply to the limbs is often impaired or temporarily stopped (hypoperfusion, ischemia), resulting in ischemia-reperfusion (I/R) injury associated with significant morbidity and mortality [1,2,3,4]. The incidence is estimated at 1.5 cases per 10,000 people [1]. The I/R injury is also a major problem in emergency care. It is very important to reduce the time of ischemia to prevent further organ damage. Indirect tissue damage may be exacerbated by shock or resuscitation. Post-cardiac arrest syndrome can also be considered part of systemic I/R injury, which leads to organ and brain failure, death. Systemic ischemia is associated with hypoxia and a lack of energy at the tissue and cellular level. In case of contradictory but successful resuscitation, recurrent circulation may further aggravate organ failure [4,5,6]. Repeated periods of ischemia and reperfusion may also cause wound healing disorders [7].

It is essential to restore circulation as soon as possible within the ischemic tolerance time of the given organ or tissue. The critical ischemic time for human muscle tissue is about 2.25 h in warm ischemia, and irreversible muscle damage starts after 3 h of ischemia and is nearly complete at 6 h [8,9]. The restoration of blood supply can further aggravate the ischemic damage and results in endothelial and parenchymal injury, referred to as I/R injury. The etiological factors include Ca^2+^ overload, oxidative stress, leukocyte infiltration, release of inflammatory mediators, endothelial dysfunction, and complement activation [9,10,11].

To prevent or reduce I/R injury, considerable effort has been made in developing various therapeutic strategies, including pharmacological and surgical approaches [3,4]. A simple strategy is hypothermia and the use of chilled heparin saline [6]. During surgeries, I/R damage can be reduced by graduate reperfusion [12]. Ischemic pre-conditioning has emerged as a powerful experimental method in decreasing ischemic injury by Murry et al. in 1986 in canine myocardium [13]. The method consists of inducing brief ischemic insults to the target organ before the subsequent prolonged ischemia. Its beneficial effect was proven in several organs and tissues [14,15]. However, the clinical application is limited due to the unpredictable onset of an ischemic insult or embolic event. A further development of the method is the remote ischemic pre-conditioning, which was described by Przyklenk in 1993 [16]. It can increase the tolerance against I/R injury in many organs, involving the brain, heart, and kidney [17]. It is based on intermittent short-term interruptions of the blood flow of another organ or extremity before the ischemic period of the target organ. The limitation of the method is that it can be applied only in case of elective interventions. The mechanism of post-conditioning was introduced by Zhao et al. [18]. In contrast to pre-conditioning, the brief periods of ischemia and reperfusion are applied at the onset of the reperfusion, after the target organ ischemia. This technique can be easily applied to the ischemic tissue after the surgery.

The red blood cell deformability and aggregation show significant changes in many pathophysiological conditions. Ischemia-reperfusion injury may cause micro-rheological changes due to metabolic changes, free radical reactions, and acute phase reactions [19,20,21]. Although a huge number of studies have examined the consequences of I/R injury to skeletal muscle and the protective effect of pre- and post-conditioning [22,23,24], very few studies have addressed the changes in the micro-rheological parameters [25,26].

In our previous studies, we have found significant alterations in blood rheological parameters due to limb ischemia and reperfusion, however, the magnitude of changes was different. The most obvious alterations were found after 3 h of ischemia (vascular clamp combined with tourniquet) that led to histological changes and rheological deterioration together. The morphological changes could be sustained by local cooling, but not the rheological ones [27]. In rat models of 1- and 2-h ischemia and following reperfusion, microrheological deterioration was found in the early reperfusion period (the first hour of reperfusion) and in a second wave on the first to third postoperative days [28,29]. The time factor is important, depending on the ischemic tolerance of the tissues. The skin and muscles are known to have relatively wide ischemic tolerance. However, it is important to note that the endothelium is very sensitive to hypoxia, and endothelial dysfunction may occur in a short time. Kayar et al. found that even 15 min of ischemia may lead to endothelial dysfunction and rheological alterations in rats [19]. So, the picture is very complex and it is not clear where the boundary of reversible and irreversible changes is. It is not known how large hemorheological change leads to a perfusion problem, and how depressed should the perfusion be to result in further changes in the tissues, not talking about the duration, temperature, and extension of complete ischemia.

The present study was therefore designed to evaluate the alterations in the hematological and micro-rheological parameters due to lower limb ischemia and to compare the expected favorable effect of pre- and post-conditioning in a rat model.

## 2. Results

### 2.1. Hematological Parameters

Hematological parameters are shown in Table 1. The white blood cell count increased just after the 120-min ischemia in the I/R (*p* = 0.018 vs. base) and in the PreC groups (*p* < 0.001 vs. base) and was elevated further by the end of the first postoperative (p.o.) week, reaching a significant level in the PreC (*p* < 0.001 vs. base) and the PostC groups (*p* = 0.008 vs. base).

Red blood cell count, hemoglobin, and hematocrit values showed a moderate decrease at the beginning of the reperfusion versus base values (RBC in I/R group: *p* = 0.021, in PreC group: *p* < 0.001; Hgb in I/R group: *p* = 0.009, in PreC group: *p* < 0.001, in PostC group: *p* = 0.004). A further decrease was observed by the first *p*.o. week (RBC, Hgb and Hct in I/R, PreC and PostC groups: *p* < 0.001). In the PostC groups, reduction of hemoglobin and hematocrit (*p* = 0.001 for both), as well as hematocrit decrease in I/R group was found to be significant compared to the Control group (*p* < 0.001).

Platelet count showed lower values after the 120-min ischemia compared to base values (Control: *p* < 0.001, I/R: *p* = 0.041, PreC: *p* = 0.002, PostC: *p* = 0.001), and rose by the first p.o. week (Control: *p* < 0.001, I/R: *p* < 0.001, PreC: *p* = 0.032, PostC: *p* < 0.001). The values of the I/R group were higher versus the Control group at the beginning of the reperfusion (*p* = 0.002) and on the first p.o. week (*p* = 0.006). The highest values were found in the PostC group one week after surgery (*p* < 0.001 vs. Control).

### 2.2. Red Blood Cell Deformability

**Figure 1:**
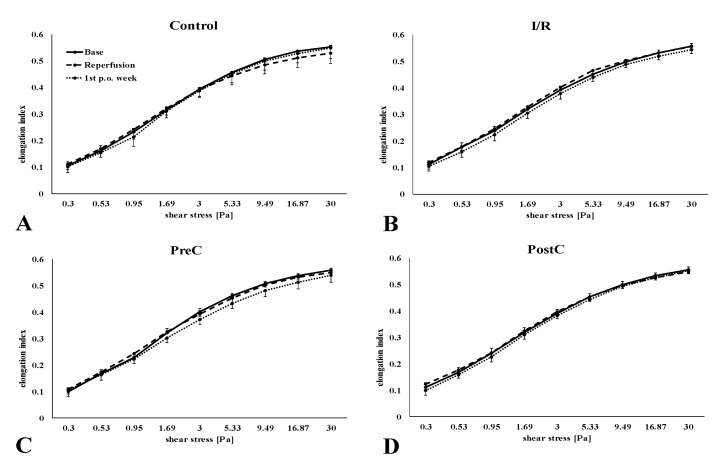
Changes of red blood cell deformability (elongation index in the function of shear stress in the control (**A**), the ischemia-reperfusion (I/R) (**B**), the ischemic pre-conditioning (PreC) (**C**) and the ischemic post-conditioning (PostC) (**D**) groups. Means ± S.D.

The cumulative elongation index (EI)—shear stress (SS) curves are shown on Figure 1, the comparative parameterization data are summarized in Table 2.

**Figure 2:**
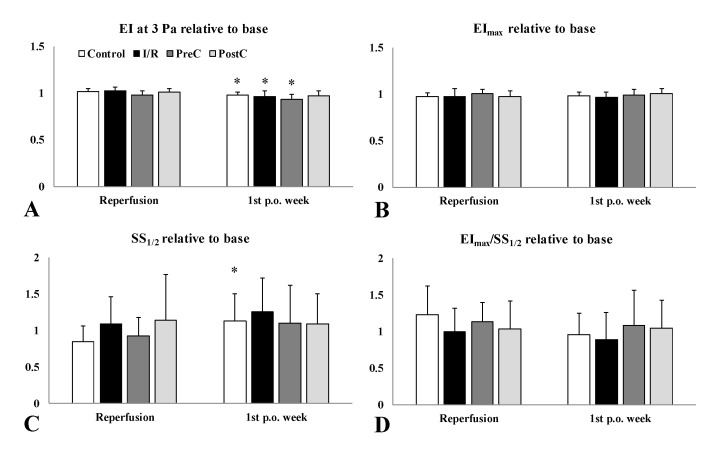
Relative values of elongation index (EI) at 3 Pa (**A**), maximal elongation index (EI_max_) (**B**), shear stress at half EI_max_ (SS_1/2_) (**C**), and the ratio of EI_max_ and SS_1/2_ (**D**), compared to their base values (as 100%) in the control, the ischemia-reperfusion (I/R), the ischemic pre-conditioning (PreC) and the ischemic post-conditioning (PostC) groups. Means ± S.D., * *p* < 0.05 vs. reperfusion.

The EI values were lower one week after surgery in the I/R group, and more obviously in the PreC group (Figure 1B,C). However, the differences were small. By the 1st p.o. week EI values at 3 Pa showed significant lowering in the I/R group compared to their base (*p* = 0.048). When analyzing the changes compared to base values, individually, these relative values revealed more differences (Figure 2). The magnitude of lowering in EI at 3 Pa was significant one week after surgery compared to the post-ischemic relative values (Control: *p* = 0.012, I/R: *p* = 0.005, PreC: *p* = 0.025). The rise in SS_1/2_ was the highest in the I/R group, which was reflected in the EI_max_/SS_1/2_ ratio (*p* = 0.002) (Figure 2).

### 2.3. Red Blood Cell Aggregation

**Figure 3:**
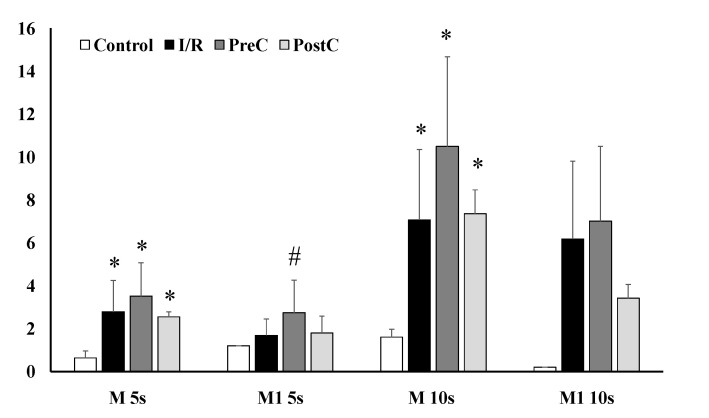
Red blood cell aggregation index values (M 5s, M1 5s, M 10s, M1 10s) in the control, the ischemia-reperfusion (I/R), the ischemic pre-conditioning (PreC), and the ischemic post-conditioning (PostC) groups on the first postoperative week. Means ± S.D., * *p* < 0.05 vs. Control group and # *p* < 0.05 vs. I/R group (same time).

Enhanced red blood cell aggregation was found in the ischemic groups (I/R, PreC, PostC) one week after surgery, showing increased M and M1 index values (Figure 3). The I/R and PreC groups expressed the highest values of M 5s (*p* < 0.001 vs. base for both), M1 5s (only in PreC group: *p* = 0.038 vs. base and *p* = 0.046 vs. Control), as well as in M 10s (*p* < 0.001 vs. base for both) and M1 10s index values (*p* < 0.001 vs. base for both) (Figure 3).

### 2.4. Blood Gases, Acid-Base Parameters, Electrolytes, and Metabolites

Table 3 summarizes the changes of blood gas, pH, electrolyte, and metabolic parameters. 

The values of *p*O_2_, *p*CO_2_ did not change significantly. The pH decreased in the PreC and PostC groups at the start of the reperfusion and normalized by the first p.o. week.

Sodium, calcium, and chloride ion concentrations did not show significant changes. Just after the ischemia, the potassium ion concentration increased significantly in all groups versus the base, with larger magnitude in the groups subjected to ischemia (Control: *p* = 0.01, I/R: *p* < 0.001, PreC: *p* = 0.011, PostC: *p* < 0.001 vs. base). By the first p.o. week, the potassium concentration was lower in I/R and in PostC groups compared to the Control (*p* = 0.007 and *p* = 0.004, respectively).

An increase was observed in the glucose concentration in all groups with ischemia and reperfusion. The rise was significant in the PostC group (*p* = 0.002 vs. base, *p* = 0.039 vs. Control). By the first p.o. week these values significantly decreased in these groups compared to base (I/R: *p*= 0.001, PreC: *p* = 0.001, PostC: *p* = 0.006) and compared to the Control group (I/R: *p*= 0.01, PreC: *p* = 0.004, PostC: *p* = 0.013). Lactate concentration increased significantly by the first p.o. week in the I/R (*p* = 0.019), PreC (*p* = 0.037) and PostC (*p* = 0.041) groups compared to the Control. Creatinine concentration increased significantly only in the PreC (*p* = 0.002 vs. base) and PostC (*p* = 0.017 vs. base, *p* = 0.008 vs. Control, *p* = 0.038 vs. I/R) groups just after the reperfusion. 

### 2.5. Histology

**Figure 4:**
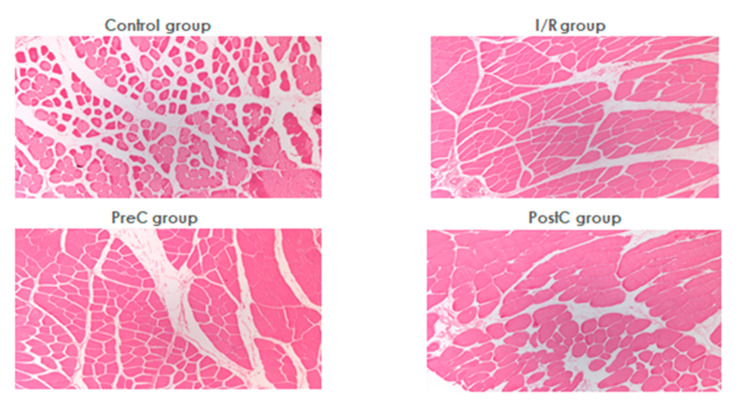
Representative histological slides of the skeletal muscle biopsies in the control, the ischemia-reperfusion (I/R), the ischemic pre-conditioning (PreC), and the ischemic post-conditioning (PostC) groups. Staining: H&E; original magnification: 50×.

No histological abnormalities attributable to ischemia were seen in any of the groups by light microscopy (Figure 4). Analyzing the postoperative samples preserved striation, regular contour of the muscle fibers was found with the normal distribution of the nuclei without any sign of hypertrophy. No disorganization of myofibrils occurred in the sarcoplasm. Swelling and intense inflammatory infiltration could not be demonstrated. Necrotic muscle fiber sections were not present and no caliber fluctuation was detected. In a pre-conditioned animal, signs of subacute inflammation and fresh bleeding were seen in the perimysium, which may have developed due to the tourniquet compression.

## 3. Discussion

The tourniquet is used in many areas of surgery, such as traumatology, orthopedics, and vascular surgery. In experimental studies, several strategies have been developed to reduce I/R injury, but very few of them have been introduced into clinical practice. Among surgical approaches, pre- and post-conditioning are promising methods [13,14,15,16,17,18], although there are still many unanswered questions involving the optimal timing of the stimulus and the number and duration of the cycles, etc. It should also be taken into account that the applicability of pre-conditioning in clinical practice is limited to scheduled surgery, while ischemic post-conditioning can be easily applied even in emergency situations [1,2,3,4,6].

To answer all questions, it is essential to know the functional and structural changes accompanied by I/R injury. Reviewing the literature, little data is available on changes in the micro-rheological parameters in relation to ischemia-reperfusion injury of extremities and ischemic conditioning surgical maneuvers.

Various models are known for unilateral hind limb ischemia, operating with vascular microvascular clips for clamping the femoral artery, tourniquet or inflated cuff around the thigh [19,27,28,29,30,31]. It is important to note that in rats, remarkable collaterals are existing from the gluteal region [30]. It has been demonstrated that clamping the femoral vessels alone often does not lead to complete ischemia. The tourniquet may compress collaterals as well, but the force applied may cause extended tissue damage. Most of the ischemic pre- and post-conditioning protocols use three or four cycles, the duration of which ranges from 10, 15 and 30 s to 10 min [13,14,18,21,22,24,32]. We have chosen 120-min tourniquet-induced ischemia, preceded or followed by three cycles or 10-min ischemia and reperfusion.

Red blood cell deformability and aggregation show significant changes in many pathophysiological conditions, including ischemia-reperfusion injury. These are mainly due to free radical reactions, metabolic changes and acute phase reactions [20,21,33,34,35,36,37]. Free radicals may damage the red blood cells by lipid peroxidation, methemoglobin formation, as well as by damaging proteins via sulfhydryl cross-linking [19,20,21]. Metabolic changes may alter the morphological and mechanical properties of red blood cells, which leads to deterioration of red blood cell deformability and disturbed aggregation [21,36]. Acute phase reactions may manifest as a rise in leukocyte count, increase or decrease of platelet count, hemoconcentration, and micro-rheological changes [20,21,34]. Impaired red blood cell deformability and enhanced red blood cell aggregation elevate blood viscosity, increase vascular resistance, and cause perfusion problem in the microcirculatory bed [20,21,38,39,40,41].

In this observational study, we found that hemoglobin and hematocrit decreased significantly after reperfusion and one week post-operatively in all ischemic groups. In parallel, the platelet count significantly increased. These changes may be associated with inflammatory processes and acute phase reactions induced by ischemia-reperfusion. The decrease in these values is due not only to I/R damage but also to blood loss caused by surgery and blood sampling. We supposed that the alterations observed post-ischemically are mostly due to redistribution changes, and the later alterations can be originated dominantly from the inflammatory processes. 

Similar to our previous findings in other ischemia-reperfusion models [25,26], micro-rheological parameters have deteriorated during and after ischemia in all ischemic groups. Red blood cell aggregation has significantly increased, mostly due to the increased free radical release, acute phase reactions, and inflammatory processes. Interestingly, the most significant increase was found in the PreC group.

Metabolic changes alter the morphological and mechanical properties of blood cells that may result in the deterioration of red blood cells’ deformability and disturbed aggregation [20,21,35,36]. Deoxygenated red blood cells have decreased deformability and enhanced aggregation [37], while hypoxia leads to swelling of the cells, altering the cellular surface/volume ratio, and thus the deformability as well [21,36]. In our experiment, the changes in metabolic and micro-rheological parameters were not clearly observed at the same time (post-ischemic vs. first p.o. week values). Therefore, also considering the low sample size, a multivariate regression analysis could not be performed. It is also noted that mathematically significant changes could be detected, but the real in vivo significance of the magnitude in changes of micro-rheological variables are still controversial. It is still not known where the border of reversible and irreversible changes is seen, and it is still obscure whether which magnitude of red blood cell deformability impairment and/or erythrocyte aggregation enhancement causes perfusion problems [21,38,39,40,41].

During ischemia, changes in mitochondrial function, enzyme activity, and ion transport may occur. ATP is rapidly dephosphorylated and converted to AMP, which is further degraded. The ion balance of the cells is upset and the intracellular concentration of H^+^, Na^+^, and Ca^2+^ increases. In the process, free radicals are formed, which also cause damage to enzymes, proteins, carbohydrates, membrane lipids, and DNA. Components released from dead cells initiate inflammatory processes and release cytokines (TNFα, IL-1β, IL-6). Inflammatory processes may be generated and systemic inflammatory response syndrome (SIRS) may develop [9,10]. We harvested tissue biopsies for histological analysis, where no obvious differences were seen between the groups. The compression we used did not cause early complications. However, histological signs of I/R damage were observed in similar animal experiments performed at our institute. Presumably, excellent collateral circulation in the lower limb of the rat [30] reduced the degree of I/R damage we caused to such an extent that, although laboratory abnormalities developed, no histologically detected damage occurred. Ischemia laser Doppler tests confirmed the decreased microcirculatory values on the toe, but not zero values. It is supposed that the applied tourniquet did not cause complete ischemia, only hypoperfusion. The strength of the tourniquet also influences its effect, however, too strong of a compression may cause direct tissue injury, which we wanted to avoid in this model. This is a limitation of the study. We also wished to investigate the possible direct damaging effect of tourniquet applications, therefore, we got biopsies from the thigh muscles. However, in respect of the collaterals [30], examining muscles at the lower region (e.g., plantar flexor complex) would be a better choice for further future studies.

Overviewing the findings, in this study we could see that at the early reperfusion period, significant metabolic and micro-rheological changes occurred. However, their magnitude was not enough to result in visible histological alteration on standard H&E sections. Ischemic pre-conditioning resulted in larger micro-rheological alterations than the post-conditioning protocol. These findings with post-conditioning are comparable to those of other research groups [24,32], however, the issue is still controversial. Some studies have shown its protective effect [23], but enhancement of the damage was also described [42].

Our study used healthy animals without any co-morbidities, with an intact vascular system and normovolemia. Although minimal changes have been observed without significant consequences, they had the potential to have more serious reactions. The effect of the slight changes may add up and be more significant if associated with pathological conditions (arteriosclerosis, bleeding, shock, malnutrition, etc.). There is no perfect model to study all of these aspects. All the animal studies have their own limitations, and numerous factors have to be taken into consideration when planning, conducting the studies and evaluating and extrapolating the results [31]. However, in a human study, we have found comparable results with this recent experimental study, when red blood cell deformability decreased and erythrocyte aggregation enhanced after ischemia-reperfusion by the first and second p.o. days, and non-steroid anti-inflammatory drug administration or ischemic pre-conditioning could moderate the changes in patients with lower extremity operations [43]. 

## 4. Materials and Methods

### 4.1. Experimental Animals

All procedures were approved and registered by the University of Debrecen Committee of Animal Welfare (permission registration Nr.: 25/2016. UDCAW) in accordance with national and EU regulations (Hungarian Animal Protection Act (Law XVIII/1998) and Directive 2010/63/EU). Thirty 8-week male Crl:WI rats were included in the experiment, and were kept in standard cages in alternating day and night light conditions in a 12-h cycle. We provided them with free access to drinking water and conventional rodent chow.

### 4.2. Operative Techniques and Sampling Protocol

The rats were anesthetized with i.p. injection of ketamine hydrochloride (100 mg/bwkg, CP-Ketamin) and xylazine (10 mg/bwkg, CP-Xylazin), combined with atropine sulfuricum (0.05 mg/bwkg). To maintain anesthesia, one third of the initial dose was administered during the procedure.

The right common carotid artery was cannulated for monitoring blood pressure in all animals. The animals were randomly divided into four groups:

I. Control (C) group (*n* = 8, 320.4 ± 9 g): besides the common carotid artery cannulation, no other intervention was performed;
II. Ischemia-reperfusion (I/R) group (*n* = 7, 376.4 ± 42.4 g): unilateral hind limb ischemia was induced by tourniquet application around the thigh, below the right inguinal region. After 120-min ischemia the tourniquet was completely released to allow full reperfusion;
III. Pre-conditioned (PreC) group (*n* = 8, 388.6 ± 39.1 g): three cycles of 10-min ischemia and reperfusion (by tightening then releasing the tourniquet, alternately) was applied before the prolonged ischemia, as described in the I/R group;
IV. Post-conditioned (PostC) group (*n* = 7, 386.7 ± 46 g): the same three cycles of ischemia-reperfusion were introduced at the onset of the reperfusion, after 120-min ischemia as described in the I/R group.

The animals received flunixin meglumine s.c. (10 mg/bwkg) postoperatively.

A laser Doppler device (LD-01 Laser Doppler Tissue Flowmeter, Experimetria Co., Budapest, Hungary; with standard pencil probe MNP100XP, Oxford Optronix Ltd., Abingdon, UK) was used to test microcirculation during the ischemia to confirm ischemia or hypoperfusion. The pencil probe was taken on the skin of the right toe. Artifact-free 20-s recordings were analyzed in each animal. Blood perfusion unit values did not drop to zero, but decreased during tourniquet application versus base values (I/R group: 77.3 ± 19.2%; PreC group: 71.1 ± 9.4%; PostC group: 71 ± 11.3%).

During the experiment, blood samples were taken from the lateral tail vein at the beginning of the procedure (before the 120-min ischemia, as Base), then after the removal of the tourniquet at the beginning of reperfusion, in group IV after the post-conditioning, and one week later in all groups. In the Control group, the timing of the second blood sampling was set 120 min after the preparation and cannulation. Occasionally, 0.3 to 0.5 mL of blood was taken (anticoagulant: 1.8 mg/mL K_3_-EDTA). Histological samples were taken from the ischemic muscle one week after the intervention.

### 4.3. Laboratory Methods

Hematological parameters were determined by a Sysmex K-4500 microcell counter (TOA Medical Electronics Co., Ltd., Kobe, Japan). Red blood cell count (RBC [×10^6^/L]), hematocrit (Hct [%]), hemoglobin (Hgb [g/dL]), white blood cell count (WBC [×10^3^/L]), and platelet count (Plt [×10^3^/L]) were analyzed in this study.

A LoRRca MaxSis Osmoscan (RR Mechatronics BV, Zwaag, The Netherlands) ektacytometer was used to test red blood cell deformability, determining the elongation index (EI [au]) in the function of shear stress (SS [Pa]) [44,45]. For the tests polyvinylpyrrolidone (PVP), normal phosphate buffered saline (PBS) solution was prepared (PVP: 360 kDa, Sigma-Aldrich Co., St. Luis, MO, USA; PVP-PBS solution viscosity = 29.5 m Pas, osmolality = 300 mOsmol/kg, pH = 7.2). For comparison, the EI values at 3 Pa of shear stress, and by parameterization of EI-SS curves (Lineweaver-Burk equation), maximal elongation index (EI_max_), the shear stress belonging to the half EI_max_ (SS_1/2_, [Pa]), and their ratio were calculated [46].

To measure red blood cell aggregation in whole blood, we used a Myrenne MA-1 erythrocyte aggregometer (Myrenne GmbH, Roetgen, Germany), based on the light-transmission principle [44,45]. Aggregation was tested in M (shear rate = 0 s^−1^) and M1 modes (shear rate = 3 s^−1^) at the 5th and 10th seconds, accordingly, the M 5s, M 10s, M1 5s and M1 10s index parameters were determined. 

An epoc^®^ Blood Analysis System (Siemens Healthineers, Erlangen, Germany) was used to test blood partial oxygen and carbon-dioxide tensions (*p*O_2_, *p*CO_2_ [mmHg]), blood pH, electrolytes (Na^+^, K^+^, Ca^2+^ and Cl^−^) and metabolites (glucose [mmol/L], lactate [mmol/L], creatinine [μmol/L]). The test card (per sample) required 0.1 mL of native blood.

### 4.4. Histology

One week postoperatively, tissue samples were taken from the biceps femoris muscle under general anesthesia. The samples were fixed in 5% formaldehyde, embedded in paraffin, microtomed into 3–5 µm sections, stained with hematoxylin and eosin (H&E), and evaluated under an optical microscope.

### 4.5. Statistical Analysis

Data are expressed as means ± standard deviation (S.D.). In case of variables when the base values showed high variety, we also analyzed the ratio of changes (relative values vs. its own base in each case). A GraphPad Prism software for Windows, version 8.0 (GraphPad Software Inc., La Jolla, CA, USA) was used for statistical analysis. Differences within and between the groups were analyzed by two-way ANOVA followed by post-hoc Bonferroni-test or Dunn’s method, depending on the result of normality test. Probability values (*p*) less than 0.05 were considered as statistically significantly different.

## 5. Conclusions

In conclusion, 2-h tourniquet-induced hind limb ischemia and reperfusion caused impairment in red blood cell deformability with enhanced erythrocyte aggregation, accompanied by hematological and metabolic changes promptly after ischemia. Ischemic pre- and post-conditioning (3 × 10-min ischemia with 10-min reperfusion periods prior or after 120-min ischemia) resulted in additive changes of various manners. Post-conditioning showed better micro-rheological effects. However, by these parameters, it cannot be clearly decided which ischemic conditioning protocol is better. Using a longer limb ischemic period (3 or 4 h, with, e.g., vascular clamping plus tourniquet application) would be recommended to use for better studying this issue.

## Figures and Tables

**Table 1:** Selected hematological parameters in the control, the ischemia-reperfusion (I/R), the ischemic pre-conditioning (PreC), and the ischemic post-conditioning (PostC) groups.

Variable	Group	Base	Reperfusion	1st p.o. Week
WBC [G/L]	Control	9.36 ± 1.85	8.62 ± 2.35	10.4 ± 3.59
	I/R	8.05 ± 1.23	10.77 ± 4.23 *	10.17 ± 4.47
	PreC	7.08 ± 1.11	10.67 ± 2.53 *	11.2 ± 4.15 *
	PostC	7.51 ± 1.57	8.77 ± 3.67	10.65 ± 3.1 *
RBC [T/L]	Control	7.78 ± 0.47	7.49 ± 0.41	7.44 ± 0.37
	I/R	8.38 ± 0.64	7.69 ± 0.71 *	7.09 ± 0.28 *
	PreC	8.35 ± 0.39	7.65 ± 0.56 *	6.93 ± 1.37 *
	PostC	8.18 ± 0.73	7.69 ± 0.77	7.09 ± 0.43 *
Hgb [g/dL]	Control	15.23 ± 0.74	14.89 ± 0.67	14.52 ± 0.83 *
	I/R	15.61 ± 0.82	14.75 ± 0.76 *	13.41 ± 0.51 *
	PreC	15.18 ± 0.41	14.14 ± 0.79 *	12.84 ± 2.56 *
	PostC	15.16 ± 0.68	14.25 ± 0.84 *	13.25 ± 0.78 * #
Hct [%]	Control	46.68 ± 2.45	45.23 ± 2.43	43.6 ± 2.07 *
	I/R	47.4 ± 3.07	44.6 ± 3.49	40.39 ± 1.31 * #
	PreC	46.7 ± 1.41	43.81 ± 2.99	38.96 ± 7.56 *
	PostC	45.93 ± 2.87	44.13 ± 3.16	40.21 ± 2.09 * #
Plt [G/L]	Control	741.2 ± 62.1	626.8 ± 99.5 *	833.6 ± 40.9 *
	I/R	810.1 ± 72.9	747.6 ± 80.5 * #	958.5 ± 100.1 * #
	PreC	726.3 ± 82.7	638.3 ± 63 *	873.1 ± 315.4 *
	PostC	712.6 ± 67.2	625.7 ± 57.2 *	1000 ± 44.5 * #

Means ± S.D.; * *p* < 0.05 vs. base (same group), # *p* < 0.05 vs. control group (same time); RBC: red blood cell count; WBC: white blood cell count; Hgb: hemoglobin concentration; Hct: hematocrit; Plt: platelet count.

**Table 2:** Erythrocyte deformability values delivered from the elongation index (EI): shear stress (SS) curves in the control, the ischemia-reperfusion (I/R), the ischemic pre-conditioning (PreC), and the ischemic post-conditioning (PostC) groups.

Variable	Group	Base	Reperfusion	1st p.o. Week
EI at 3 Pa	Control	0.393 ± 0.007	0.392 ± 0.024	0.387 ± 0.01
	I/R	0.392 ± 0.013	0.402 ± 0.009	0.378 ± 0.02 *
	PreC	0.400 ± 0.012	0.392 ± 0.012	0.371 ± 0.018
	PostC	0.391 ± 0.013	0.397 ± 0.01	0.382 ± 0.012
EI_max_	Control	0.582 ± 0.015	0.553 ± 0.049	0.571 ± 0.017
	I/R	0.601 ± 0.02	0.587 ± 0.022	0.581 ± 0.032
	PreC	0.585 ± 0.019	0.589 ± 0.024	0.588 ± 0.046
	PostC	0.595 ± 0.028	0.576 ± 0.023	0.593 ± 0.022
SS_1/2_ [Pa]	Control	1.44 ± 0.25	1.2 ± 0.42	1.53 ± 0.3
	I/R	1.23 ± 0.33	1.26 ± 0.25	1.45 ± 0.27
	PreC	1.36 ± 0.25	1.21 ± 0.23	1.33 ± 0.52
	PostC	1.28 ± 0.46	1.32 ± 0.36	1.34 ± 0.25
EI_max_/SS_1/2_ [Pa^−1^]	Control	0.419 ± 0.092	0.52 ± 0.19	0.387 ± 0.087
	I/R	0.524 ± 0.156	0.488 ± 0.136	0.418 ± 0.112
	PreC	0.454 ± 0.143	0.499 ± 0.096	0.553 ± 0.365
	PostC	0.487 ± 0.223	0.473 ± 0.165	0.458 ± 0.103

Means ± S.D.; * *p* < 0.05 vs. base (same group); EI_max_: maximal EI; SS_1/2_: shear stress at half EI_max._

**Table 3:** Changes of blood gas (*p*O_2_, *p*CO_2_), pH, electrolytes (Na^+^, K^+^, Ca^2+^, Cl^−^,) and metabolites (glucose, lactate, creatinine) in the control, the ischemia-reperfusion (I/R), the ischemic pre-conditioning (PreC), and the ischemic post-conditioning (PostC) groups.

Variable	Group	Base	Reperfusion	1st p.o. Week
*p*O_2_ [mmHg]	Control	68.87 ± 13.26	65.73 ± 11.05	55.2 ± 6.93
	I/R	60.14 ± 6.61	61.37 ± 8.49	55.06 ± 9.22
	PreC	65.05 ± 5.75	69.47 ± 15.27	51.78 ± 11.8
	PostC	59.65 ± 9.27	68.08 ± 6.57	57.1 ± 16.21
*p*CO_2_ [mmHg]	Control	41.3 ± 9.04	43.55 ± 5.54	52.62 ± 11.23
	I/R	50.45 ± 6.57	49.94 ± 17.77	49.4 ± 4.72
	PreC	47.64 ± 6.78	48.01 ± 11.17	42.42 ± 4.95
	PostC	47.78 ± 6.62	47.68 ± 12.2	45.12 ± 5.86
pH	Control	7.41 ± 0.03	7.37 ± 0.03	7.36 ± 0.06
	I/R	7.36 ± 0.04	7.33 ± 0.11	7.38 ± 0.04
	PreC	7.38 ± 0.07	7.31 ± 0.07	7.41 ± 0.04
	PostC	7.35 ± 0.03	7.32 ± 0.08	7.39 ± 0.02
Na^+^ [mmol/L]	Control	142.12 ± 3.35	141.83 ± 2.13	143.6 ± 2.61
	I/R	141 ± 2.31	140.28 ± 5.4	142 ± 2.34
	PreC	142.28 ± 2.69	140.28 ± 4.46	141.71 ± 2.56
	PostC	142.42 ± 3.2	140.14 ± 2.47	143.85 ± 2.41
K^+^ [mmol/L]	Control	4.72 ± 0.32	5.45 ± 0.56 *	4.82 ± 0.29
	I/R	4.32 ± 0.28	5.92 ± 0.57 *	4.04 ± 0.38 #
	PreC	4.25 ± 0.25	5.58 ± 0.86 *	4.61 ± 0.5
	PostC	4.24 ± 0.25	6.05 ± 0.62 *	4.17 ± 0.29 #
Ca^2+^ [mmol/L]	Control	1.36 ± 0.04	1.38 ± 0.11	1.28 ± 0.19
	I/R	1.35 ± 0.04	1.39 ± 0.04	1.23 ± 0.31
	PreC	1.34 ± 0.06	1.39 ± 0.06	1.27 ± 0.17
	PostC	1.39 ± 0.05	1.39 ± 0.03	1.32 ± 0.09
Cl^−^ [mmol/L]	Control	104.25 ± 1.67	108.16 ± 3.18	104.6 ± 1.34
	I/R	103.14 ± 2.11	105.85 ± 2.19	102.6 ± 1.67
	PreC	103.14 ± 2.03	104.85 ± 2.79	104 ± 1.52
	PostC	104 ± 2.31	106.28 ± 1.6	103.85 ± 2.19
glucose [mmol/L]	Control	19.62 ± 3.54	17.11 ± 3.47	17.94 ± 3.08
	I/R	17.98 ± 2.09	19.6 ± 4.27	12.34 ± 2.21 * #
	PreC	17.22 ± 2.19	22.48 ± 7.61	11.8 ± 2.61 * #
	PostC	16.5 ± 1.09	22.64 ± 4.77 * #	13.12 ± 2.45 * #
lactate [mmol/L]	Control	1.171 ± 0.34	1.52 ± 0.61	1.02 ± 0.32
	I/R	1.61 ± 0.73	1.36 ± 0.34	2.31 ± 0.91 #
	PreC	1.78 ± 0.87	1.72 ± 0.85	3.11 ± 1.89 #
	PostC	1.82 ± 0.99	1.28 ± 0.31	3.39 ± 2.2 #
creatinine [µmol/L]	Control	31 ± 4.37	43.83 ± 10	36.2 ± 3.89
	I/R	35.33 ± 5.68	47.85 ± 8.47	37.2 ± 5.26
	PreC	32.42 ± 3.59	68.42 ± 31.28 *	52.85 ± 25.51
	PostC	43.85 ± 8.45	83 ± 43.77 * # +	35.71 ± 6.15

Means ± S.D.; * *p* < 0.05 vs. base (the same group), # *p* < 0.05 vs. Control group and + *p* < 0.05 vs. I/R group (at the same time).

## Data Availability

The data presented in this study are available on request from the corresponding author.
